# Endocrine disrupter effects on the gonads and pubertY

**DOI:** 10.1186/1687-9856-2013-S1-O8

**Published:** 2013-10-03

**Authors:** Olle Söder

**Affiliations:** 1 Department of Woman and Child Health; Paediatric Endocrinology Unit; Karolinska Institutet & University Hospital, Stockholm, Sweden

## 

Evidence accumulating the past decades indicates that human populations are exposed to environmental chemicals interfering with endocrine systems. There is growing concern of the potential adverse impact of exposure to such endocrine disrupting chemicals (EDCs) on human health, based on observations in wildlife, animal model systems and reports of yet unexplained increased incidences of hormone related disorders in humans. Exposure in fetal and neonatal age may be associated with developmental and reproductive disturbances due to interference with the programming of normal hormone signaling and metabolic pathways. Boys are conceived to be more vulnerable than girls due to their more complicated and androgen driven prenatal sex differentiation.

Male prenatal sex differentiation is crucially dependent on the functioning of fetal Leydig cells. Exposure of these cells to EDCs at critical time windows of development may result in undermasculinization and disorder(s) of sex development (DSD), such as hypospadias, cryptorchidism and ambiguous genitalia. Furthermore, a novel trend of earlier start of puberty has been found during the past few decades, at least in some regions and mostly affecting girls. Among plausible causes behind this phenomenon it has been suggested that exposure to EDCs affecting the programming or triggering of the pubertal clock could result in premature activation of gonadotropin secretion. Another hypothesis is that gonadotropin independent effects of EDCs are associated with an earlier start of puberty.

Proof of principle of such potential roles of EDCs has been obtained from a multitude of studies in experimental animals and gained some support by case studies, exposure data and epidemiological investigations in humans. This presentation will review recent data and ongoing studies on the mechanism(s) of action of EDCs, including effects of mixtures, on the hypothalamus- pituitary-gonadal axis in experimental animals and humans. The impact of genetic susceptibility on the degree of disrupting activity caused by certain EDCs will be discussed on the basis of ongoing animal studies.

